# Recent Advances on Multivalued Logic Gates: A Materials Perspective

**DOI:** 10.1002/advs.202004216

**Published:** 2021-02-26

**Authors:** Sae Byeok Jo, Joohoon Kang, Jeong Ho Cho

**Affiliations:** ^1^ Department of Chemical and Biomolecular Engineering Yonsei University Seoul 03722 South Korea; ^2^ School of Advanced Materials Science and Engineering Sungkyunkwan University (SKKU) Suwon 16419 Republic of Korea

**Keywords:** graphene, Moore's law, multivalued logic, negative differential resistances, organic semiconductors, quantum dots, transition metal dichalcogenides

## Abstract

The recent advancements in multivalued logic gates represent a rapid paradigm shift in semiconductor technology toward a new era of hyper Moore's law. Particularly, the significant evolution of materials is guiding multivalued logic systems toward a breakthrough gradually, whereby they are transcending the limits of conventional binary logic systems in terms of all the essential figures of merit, i.e., power dissipation, operating speed, circuit complexity, and, of course, the level of the integration. In this review, recent advances in the field of multivalued logic gates based on emerging materials to provide a comprehensive guideline for possible future research directions are reviewed. First, an overview of the design criteria and figures of merit for multivalued logic gates is presented, and then advancements in various emerging nanostructured materials—ranging from 0D quantum dots to multidimensional heterostructures—are summarized and these materials in terms of device design criteria are assessed. The current technological challenges and prospects of multivalued logic devices are also addressed and major research trends are elucidated.

## Graphical abstract

The recent progress in multivalued logic gates based on emerging materials signifies a paradigm shift in the electronics technology toward the new era of hyper Moore's law. This comprehensive report reviews the evolution of emerging materials toward the realization of multivalued logic operations and highlights the challenges and prospects and elucidates major research trends.

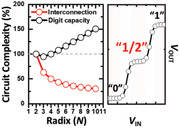

## 1 Introduction

**Figure 1:**
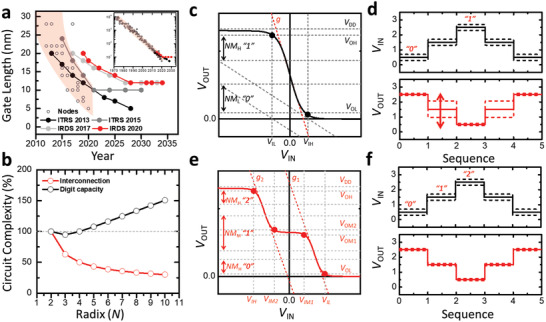
a) Projected advancements in semiconductor technology with respect to physical gate lengths for high performance logic reported in ITRS 2013, ITRS 2015, IRDS 2017 and IRDS 2020. Open circles represent the technological nodes practically realized until 2020. The inset shows the full scale view since 1970. b) Theoretical estimation of circuit complexity as a function of radix of operation. Typical input/output characteristics and figures of merit for c) binary inverter and e) STI. Temporal responses of d) binary inverter and f) STI under input voltage fluctuations.

The conventional von Neumann architecture, in which computing and memory units are physically separate, is an essential element of the current state‐of‐the‐art information processing technology. The unit device of this computing system follows Moore's law, which indicates that the number of devices and the level of the integration have historically doubled every 2 years.^[^ ^1^, ^2^, ^3^, ^4^ ^]^ However, there are physical limitations on the downscaling of device dimensions despite the high demand for performance improvement of devices in applications that involve processing of huge amounts of data, e.g., machine learning, artificial intelligence, and Internet of Things. Many technological advancements have been targeted toward the development of area‐efficient and energy‐efficient architectures with the ultimate aim of overcoming these limitations. For example, extreme ultraviolet (EUV) photolithography using the EUV wavelength of 13.5 nm enables sub‐10‐nm patterning.^[^ ^5^, ^6^, ^7^ ^]^ New device structures for achieving high computing performance, such as gate‐all‐around field‐effect transistors (FETs),^[^ ^8^, ^9^, ^10^, ^11^ ^]^ silicon‐on‐insulator (SOI) structures,^[^ ^12^, ^13^, ^14^, ^15^, ^16^ ^]^ and fin‐FET devices,^[^ ^17^, ^18^, ^19^, ^20^ ^]^ have been proposed. Although these approaches temporarily reinstate Moore's law, next‐generation computing systems are moving into a new era of hyper Moore's law.^[^ ^21^ ^]^ **Figure** **1**a shows the progress and prospects of the semiconductor technology as outlined in the 2013 and 2015 reports of the International Technology Roadmap for Semiconductors (ITRS) and in the 2017 and 2020 reports of the International Roadmap for Devices and Systems (IRDS). The semiconductor technology has thus far been evolving according to the projections made by the ITRS, but it is now at a crossroads of saturating or achieving a breakthrough to evolve further in the next 10 years. Thus, researchers are seeking alternatives to the conventional von Neumann architecture, in which computations can be performed using computing units that are not physically separate from memory units; such technology is termed in‐memory computing.^[^ ^22^, ^23^, ^24^, ^25^, ^26^, ^27^, ^28^, ^29^, ^30^, ^31^ ^]^ However, technologies that are more tangible and plausible and capable of surpassing the computing performance of the conventional von Neumann architecture are also available; these technologies can serve as a platform for increasing the information density per given unit device using ternary, quaternary, or even higher multivalued logic (MVL) systems.^[^ ^15^, ^16^, ^32^, ^33^, ^34^ ^]^ Current processing systems are based on the binary system, wherein information is stored as a “0” bit or a “1” bit; in contrast, the MVL system functions as a ternary, quaternary, or even higher‐valued system, which enables significant reductions in the number of devices and the overall system complexity. For instance, system complexity can be drastically reduced to 63% by shifting from the binary to the ternary logic system and further reduced down to 30% by increasing the number of logic states to 10^[^ ^32^ ^]^ (Figure 1b).

To this end, tremendous efforts have been made for realizing MVL gates by circuit‐level approaches. From the early 1970s, researchers demonstrated the feasibility of using a circuit combination of binary metal–oxide–semiconductor FETs (MOSFETs) and multiple resistors and complex circuit designs to construct various three‐valued logic gates.^[^ ^35^, ^36^, ^37^, ^38^, ^39^, ^40^, ^41^ ^]^ The inevitable high power consumption and high complexity of the integrated circuits and their consequent delayed operating speed, in turn, motivated further evolution of the technology to realize subthreshold‐voltage ternary operation. For example, a combination of enhancement‐mode and depletion‐mode complementary MOS (CMOS) devices was used to design multithreshold‐voltage logic gates such as a simple ternary inverter (STI), positive ternary inverter (PTI), and negative ternary inverter (NTI).^[^ ^42^, ^43^, ^44^, ^45^ ^]^ However, the incompatibility of these devices with the existing CMOS technology and the higher number of interconnections required in the construction of basic logic functions were major bottlenecks in ensuring the comparative superiority of these devices to the existing binary‐logic‐based technology. Naturally, the essentiality of mitigating those issues highlighted the necessity for devising de novo approaches that can provide not only high information density but also high area efficiency or energy efficiency at the device level. Component devices with multiple threshold voltages (or multiple states) in a single (or monolithic) device have emerged as a plausible solution, which can significantly lower the number of devices required to construct a logic gate in an area‐efficient manner.^[^ ^16^, ^34^, ^46^, ^47^, ^48^, ^49^, ^50^, ^51^, ^52^, ^53^, ^54^ ^]^ Hence, considerable research has been focused on achieving three or higher 1) equiprobable, 2) distinctive, and 3) stable electrical states through a reconfiguration of the device architecture or a physical manifestation of the energetic third state in the material itself. Because of the recent evolution of materials and the development of creative multicomponent device designs, an increasing number of studies have been able to demonstrate the feasibility of constructing ternary or even higher‐valued logic gates.

Despite the increasing interest in and promising results obtained for MVL gates, few studies have addressed the practical criteria for materials that can be used to realize these devices. Therefore, it has now become imperative to have a comprehensive overview on the progress of MVL gates and to provide a specific guideline for future research prospects from a materials perspective. In this review, we first present the current research status of MVL devices and systematically classify them to elucidate the major research trends and their prospects. We begin by presenting simplified design criteria for ternary logic gates and categorizing circuit‐level and device‐level approaches according to their operating principles. We then present representative device‐level approaches such as the use of quantum dot (QD)‐gated transistors, antiambipolar transistors (AATs), negative transconductance (NTC) transistors, and negative differential resistance (NDR) devices. We provide the figures of merit (FOMs) for the component devices in each approach and examine the evolution of materials toward the optimization or realization of FOMs. In particular, we summarize recent advancements in low‐dimensional materials such as QDs, nanowires, and atomically thin layered materials (e.g., graphene, black phosphorous (BP), transition metal dichalcogenides (TMDCs), and chemically versatile organic semiconductor heterostructures) in terms of their advantages and limitations in yielding ideal device properties when processed using each device‐level approach. Emerging materials that can exploit various chemical properties such as phase transitions and redox reactions are also introduced. Other promising approaches including resistive switching memristors and spintronics are briefly summarized.

## 2 Recent Advances in the Field of Multivalued Logic Gates

### 2.1 Fundamentals of Multivalued Logic Gates

MVL gates serve as a tool for increasing the information density by providing multiple states of electrical characteristics. In contrast to the conventional binary system, which consists of only a “0” bit and a “1” bit as the on‐state and off‐state, respectively, MVL gates have one or more additional intermediate states to define ternary, quaternary, or even higher‐valued systems.^[^ ^32^ ^]^ Addition of a state to a binary logic system (i.e., a ternary logic system) provides significantly high area efficiency, e.g., a reduction of more than 35% in the number of unit devices and interconnect lines. This reduction percentage increases to 50% in the case of a quaternary logic system (i.e., a system with two additional intermediate states), which requires half the number of digits to store the same amount of information as its binary equivalent.

All binary logic gates can have two logic levels (“0” and “1”) in the input and output states. Representative examples of binary gates are AND, OR, and NOT gates. The AND and OR gates have two or more inputs to produce one output, whereas the NOT gate has one input to produce one output. The rule for the AND binary gate is that if all the inputs are 1, the output is 1; otherwise, the output is 0. The OR gate yields an output of 1 when either of the inputs is 1. The NOT gate, also termed an inverter gate, has an output state that is opposite to the input state. For example, if the output is 1, the input is 0, and vice versa. A ternary logic gate has an additional intermediate state; that is, it has three input logic states, “0,” “1,” and “2”; examples of representative ternary logic gates are LET, ROT, and LIBRA. Similarly, quaternary logic gates have four logic levels (“0,” “1,” “2,” and “3”) in the input and output states. As the system moves from a binary to a ternary system or from a binary to a quaternary system, the system complexity reduces significantly to log_3_ *N*/log_2_ *N* or log_4_ *N*/log_2_ *N*, respectively, where *N* denotes the radix of the operation. Here, the term radix can be used interchangeably with the binary (radix of 2), ternary (radix of 3) and so on. However, it is necessary to point out that as *N* increases, the required operator function also diversifies. **Tables** **1** and **2** present the truth tables of the basic ternary operators. Three types of devices can convey the “inversion” operation of an inverter gate: STI, PTI, and NTI. The universal logic gates, ternary NAND and NOR gates, therefore have the same complexity as the ternary inverters according to de Morgan's law, which enables dissociation of positive and negative binary operators (NAND and NOR gates, respectively) into two positive or two negative unary operators (NOT gates).^[^ ^45^ ^]^ For simplicity, we review the operation and design of the simplest MVL gate, STI, throughout this report. The material and device criteria used in the realization of the STI can be expanded to higher‐level operators by using complex circuit‐level designs.

**Table 1:** Truth table for ternary inverter gates

Input X	STI	NTI	PTI
2	0	2	0
1	1	0	0
0	2	0	2

**Table 2:** Truth table for ternary NAND and ternary NOR universal gates

Input X	Input Y	T‐NAND	ST‐NAND	NT‐NAND	PT‐NAND	T‐NOR	ST‐NOR	NT‐NOR	PT‐NOR
2	2	2	0	0	0	2	0	0	0
2	1	2	0	0	0	1	1	0	2
2	0	2	0	0	0	0	2	2	2
1	2	2	0	0	0	1	1	0	2
1	1	1	1	0	2	1	1	0	2
1	0	1	1	0	2	0	2	2	2
0	2	2	0	0	0	0	2	2	2
0	1	1	1	0	2	0	2	2	2
0	0	0	2	2	2	0	2	2	2

In addition to information density, various FOMs such as power dissipation, operating speed, very‐large‐scale‐integration (VLSI) compatibility, and circuit complexity—which are not mutually exclusive but can be controlled separately—are used to assess MVL gates.^[^ ^32^, ^33^, ^39^ ^]^ The FOMs are dependent on the complexity of the circuit design and strongly dependent on the ideality of device operation. The basic requirements for ideal operation of MVL gates can be derived from those of the binary logic gates. Figure 1c shows the voltage transfer curve (VTC) of a binary inverter gate. The inverter gain ***g*** is defined as the rate at which the logic state shifts with respect to the input voltage, i.e., ∂*V*_OUT_/∂*V*_IN_. In an ideal operation, the gain should be infinite so that the device can have only two output states, “0” and “1.” However, in real‐life applications, the nonideality of the environmental and operating conditions compels us to set acceptable ranges of the output voltage for each logic state, which is termed the noise margin. To this end, the maximum low input voltage and the minimum high output voltage (*V*_IL_ and *V*_OH_, respectively) and the minimum high input voltage and the maximum low output voltage (*V*_IH_ and *V*_OL_, respectively) are first defined on the VTC under the unity gain condition (***g*** = −1) in high and low logic states, respectively. Then, the noise margin for the high logic state, NM_H_, is defined as NM_H_ = *V*_OH_ − *V*_IH_ and that for the low logic state, NH_L_, is defined as NH_L_ = *V*_IL_ − *V*_OL_, where both are expressed in the unit of *V*_DD_. Apparently, the noise margin increases with increasing gain; this results in a widening of the validity range of each logic state. However, ensuring a high ***g*** value will alone be insufficient to construct an ideal device. The gain ***g*** is, in general, dependent on the geometrical characteristics of the component devices as well as on *V*_DD_, the latter of which also affects the signal swing and gate delay. Ensuring a high ***g*** value can therefore enable low‐voltage operation, which, in turn, would lead to low power consumption; however, a high ***g*** value can also have adverse effects on the robustness of the signals to nonscaling external noises and the operating speed.

An input voltage corresponding the output voltage beyond the noise margin can constitute a third logic state (“1/2”) in the temporal response of a binary inverter. However, this intermediate state does not meet the basic requirements of being an equiprobable, distinctive, and stable state with respect to the low and high logic states (Figure 1d). Given the nonideality of the operational stability (e.g., bias stability) and environmental stability (e.g., degradation) during practical operations, generation of the third state is unstable. Therefore, binary inverters will not suffice for the synthesis of the ternary logic even when they are ideally optimized.

Figure 1e,f shows, respectively, a schematic VTC and the temporal response of the representative ternary inverter, i.e., the STI. FOMs similar to those of binary inverters can also be rationally applied to assess the ideality of STI performance. As is the case with binary inverters, ensuring the maximum noise margins with high gains (***g***_1_ and ***g***_2_, corresponding to the transition from the “0” logic state to the “1” logic state and that from the “1” logic state to the “2” logic state, respectively) and a high signal swing are prerequisites for achieving ideal FOMs of ternary inverters with low power consumption and high resistance to external noise (noise immunity). However, the presence of nonzero multithreshold voltages in the component transistors of STIs as well as other common ternary inverters, including the PTI (always “on”) and the NTI (always “off”), places additional importance on the reduction of power consumption via optimization of the intermediate states. In ternary inverters, which comprise three‐state component devices which are the primary focus of this review, the charge mobility, the distance between the threshold voltages (Δ *V*_th_ = *V*_th,0 → 1_  − *V*_th,1 → 2_), and the subthreshold swings (SSs) of the component transistors determine the gate voltage (*V*_G_) range viable for accessing the intermediate state and consequently the power consumption in circuits comprising those three‐state component transistors. Tuning of these parameters of component transistors to achieve a narrow input voltage (*V*_IN_) range for the intermediate state of an inverter can evidently enable a low‐voltage transition between logic states. However, it should be noted that such tuning can also limit the equiprobable generation of all three logic states. In this sense, the stability of the intermediate logic state, i.e., the equiprobable generation of a distinctive intermediate output state within finely controlled input ranges, is also considered an additional FOM specific to multivalued inverters. The operational stability, i.e., the noise margin, determines the operational reliability of the inverter under nonideal operating conditions. Insensitivity to geometric scaling (the width and length of each device) also affects the stability of the third (intermediate) state and therefore governs the feasibility of small‐scale integration of such devices.

**Figure 2:**
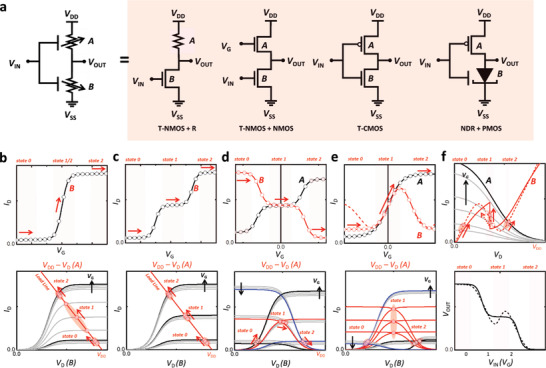
a) Representative equivalent circuits of STIs. Schematics of transfer characteristics of component devices (top) of inverters in various configurations depicted in a) and load‐line analyses of these inverters (bottom): b) NMOS + R, c) T‐NMOS + R, d) T‐CMOS, and e) T‐NMOS + NMOS configurations. f) Load‐line analysis (top) and STI characteristics (bottom) of devices in NDR + PMOS configuration. The circuit symbol for the NDR device is that of a tunnel diode. A and B in (b)–(f) correspond to the component devices of the equivalent circuits in (a). The arrows in each figure indicate the direction in which the gate voltage increases.

Now that we have established the FOMs and the corresponding design criteria for the ternary inverter, we need to examine the design criteria for the component devices (elements) of an STI that can yield the desired characteristics of the ternary inverter. Understanding the component devices can ultimately help establish design criteria for emerging materials for the ternary inverter. From the simplest standpoint, operation of the STI can be viewed in the framework of a voltage divider functioning with voltage‐controlled variable resistors.^[^ ^53^ ^]^ **Figure** **2**a shows the representative equivalent circuits of two component devices (A and B) used to realize the inverter. Depending on the turn‐on of the respective elements in the series, *V*_DD_ is distributed to a component device depending on its state (turned on or turned off) in the circuit, and this leads to variable *V*_OUT_ values. For example, when component B is completely turned off (high resistance) and component A is completely turned on (low resistance) at an input voltage corresponding to a low logic input, the logic circuit provides *V*_OUT_ ≅ *V*_DD_ (at the maximum signal swing), which corresponds to a higher logic output. A multistate ternary inverter has a half‐on state that can similarly distribute *V*_DD_ to each component to generate an intermediate logic state.

In this sense, the inverter function can be easily realized using various combinations of component elements. A linear load resistor can be used as element A and a ternary n‐type MOS (T‐NMOS) driver transistor can be used as element B (or conversely, a ternary p‐type MOS (PMOS) transistor and a linear resistor can be used as element A and element B, respectively), which is labeled the T‐NMOS + R equivalent circuit in Figure 2a.^[^ ^34^ ^]^ Similarly, the series connection of two NMOS transistors or two PMOS transistors in a non‐CMOS configuration with separate gate inputs can also be used, where transistor A functions as a bias‐controllable load resistor (T‐NMOS + NMOS).^[^ ^47^ ^]^ Ease of fabrication and high‐speed operation are possible additional advantages of this configuration. Combination of T‐PMOS and T‐NMOS transistors in a CMOS configuration can also work similarly and provide an advantage of lower power consumption (ternary CMOS (T‐CMOS))^[^ ^48^ ^]^ than other configurations. In addition to three‐state transistors, three‐state two‐terminal devices such as NDR devices can also be used to realize a ternary inverter (NDR + PMOS).^[^ ^52^ ^]^

Figure 2b–f depicts the operating mechanism of each equivalent circuit shown in Figure 2a. A simple transfer curve (*I*_D_–*V*_G_) of a binary NMOS configuration (top) and a load‐line analysis of the binary inverter based on the NMOS + R configurations (bottom) are shown in Figure 2b for easier understanding of the operating mechanism. If we consider constant current flow in a series circuit (Kirchhoff's current law), both the NMOS transistor and the resistor should show the same *I*_D_ with a division of the source voltage (*V*_DD_) into *V*_D_ and (*V*_DD_ − *V*_D_), respectively. The intersection between the two curves is therefore called the operating point of an inverter. With a change in *V*_G_ of the NMOS transistor, the operating point correspondingly moves to the transfer curve of this device, wherein the stable current levels in state “0” and state “1” of the transfer curve provide distinct semiconstant operating points for the output logic states “0” and “1” of the inverter.

As depicted in Figure 2c, a three‐state transfer curve provides three stable operating points in the load‐line analysis of a T‐NMOS + R configuration, and this, in turn, leads to the appearance of three input‐voltage‐divided logic states, i.e., “0,” “1,” and “2,” of an inverter. The selection of the load resistor (T‐NMOS + R) or the appropriate gate modulation of the load transistor (T‐NMOS + NMOS) governs the feasibility of equiprobable manifestation of the logic states and the power consumption. However, as mentioned earlier, the charge mobility, the distance between the threshold voltages (Δ*V*_th_), and the SSs between the logic states in the transfer curve of a T‐NMOS transistor itself can determine the operational stability and noise tolerance of the inverter. The power consumption should also be taken into account in the optimization of Δ*V*_th_ of the T‐NMOS transistor such that high voltages are not required to access the low (“0”) and high (“2”) logic states.

The inverter based on the combination of a T‐NMOS transistor and a T‐PMOS transistor in the CMOS configuration also has a similar operating mechanism. (Figure 2d). The transfer curves of both the NMOS transistors should show similar *V*_G_ ranges and *I*_D_ levels for the intermediate state in order to obtain a stable operating point for the “1” state. Differences in *V*_G_ ranges lead to unstable formation of the intermediate state because of the vast difference among the operating points at different *V*_G_ values, as is the case in binary inverters. A deviation in *I*_D_ values necessitates additional component elements such as resistors, which, in turn, increase the circuit complexity, power consumption, and delays in gate responses. These multithreshold transistors (T‐NMOS and T‐PMOS) can be realized and optimized by making use of materials with variable electrical/energetic states, such as doped carbon nanotubes, multiphase oxides with quantized mobility edges, and size‐controlled QDs with quantized and isolated energy levels. Details of the material criteria are provided in later sections.

We can further replace one of the three‐state transistors in the T‐CMOS configuration or T‐NMOS + NMOS configuration with another nonrectifying transistor with distinct electrical behaviors. The top panel in Figure 2e shows the transfer curves of the AAT (solid red line with empty circles), NTC ambipolar transistor (dotted red line), and n‐type transistor (solid black line with open circles), and the bottom panel shows the load‐line analysis of an inverter composed of these devices. The logic states “0” and “2” are determined by the same mechanism as that described above for multithreshold‐voltage devices. However, the intermediate state, “1,” is essentially generated via matching of their *I*–*V* slopes in transfer curves of component devices. When they share the same slope in some *V*_G_ range, the position of the input voltage at the operating point can remain unchanged within that range; this provides a stable input‐voltage‐divided logic state between “0” and “2.” The formation of heterojunctions within the channel region of a transistor through vertical stacking of p‐type and n‐type semiconductors or spatially and heavily doped graphene transistors have been proposed to induce these distinct behaviors. Detailed examples of these nonlinear component devices and their characteristics are reviewed in later sections.

Finally, a combination of an NDR device and a transistor can also be used to realize the inverter function. Instead of the use of a three‐state transistor in the NMOS + R configuration, the linear load resistor is replaced with a nonlinear one to obtain multiple stable operating points. Figure 2f shows the load‐line analysis of an STI in this configuration. As in the common NMOS + R configuration, the high and low logic states in this NDR + NMOS configuration are determined to be at the operating points at which the NMOS transistor is in the on‐state and off‐state, respectively. Between these logic states, the peak–valley structure in the current–voltage characteristics of an NDR device provides a *V*_D_ range in which a change in *V*_G_ of the NMOS transistor does not change the division of *V*_DD_ at the operating points. This behavior leads to the appearance of a *V*_IN_ (or *V*_G_)‐divided stable intermediate state in the inverter operation. Since the operating points for the intermediate state lie within the voltage range of the NDR device, the *V*_IN_ range for accessing the “1” state is determined by the peak‐to‐valley current ratio (PVCR) of the NDR device. Moreover, there is an imbalance between the operating points because of the broadening of the voltage range for the NDR device (dotted red line in the top panel), and this imbalance causes a fluctuation in the intermediate state (dotted black line in the bottom panel). In this sense, acquisition of a stable intermediate state requires not only a high PVCR but also a low NDR value itself within the NDR range. Combinations of multiple NDR devices can also be used to realize MVL gates by making use of the inherently multistable electrical characteristics under oscillating input voltages. Among the various approaches available for the realization of MVL gates, the NDR approach has been under the greatest scrutiny because the field of NDR devices—which have applications other than MVL applications—has been around for a long time and numerous chemical and physical approaches for the realization of these devices are already available. In Section 2.3, we summarize these approaches and their operating mechanisms and also present the material selection criteria for achieving NDR characteristics suitable for MVL applications. **Table** 3 summarizes approaches and corresponding materials selections for the realization of MVL gates based on the criteria introduced above.

**Table 3:** Summary of approaches for MVL gates

Class of materials	Material(s)	Device type^a)^	Logic Configuration^b)^	Radix	Operating window^c)^	FOM^d)^	Year	Ref.
Stacked bulk	In_0.53_Ga_0.47_As/AlAs/InAs	NDR (RTD)	NDR only	N/A	1–5 V	PVCR 30	1988	^[^ ^65^ ^]^
	In_0.53_Ga_0.47_As/In_0.52_Al_0.48_As/In_0.53_Ga_0.47_As	NDR (RTD)	NDR only	N/A	0–1 V	PVCR 144	1994	^[^ ^81^ ^]^
	Si/Si_0.6_Ge_0.4_/Si	NDR (RTD)	NDR only	N/A	0–1 V	PVCR 2	2003	^[^ ^83^ ^]^
	GaSb nw/InAsSb nw	NDR (ED)	NDR only	N/A	0–0.9 V	PVCR 3.5	2011	^[^ ^73^ ^]^
Stacked 2D	Graphene/BN/graphene	NDR (RTD)	NDR only	N/A	−0.5 to 0.5 V	PVCR 4	2013	^[^ ^87^ ^]^
	Three‐stacked MoS_2_	NDR (RTD)	NDR only	N/A	0–0.6 V (0.50–0.52 V)	N/A	2014	^[^ ^91^ ^]^
vdW TMDC heterostructure	MoS_2_/WSe_2_/graphene	NDR (ED)	NDR only	N/A	−1.5 to 1. 5 V (0.6–0.9 V)	PVCR 1.9	2015	^[^ ^92^ ^]^
	WSe_2_/MoSe_2_/graphene	NDR (ED)	NDR only	N/A	−1.5 to 1. 5 V (0.4–1.2 V)	PVCR 2.2	2015	^[^ ^92^ ^]^
	n‐MoS_2_/bi‐WSe_2_	NDR (ED)	NDR only	N/A	0–50 V (15–30 V)	PVCR 1.6	2015	^[^ ^94^ ^]^
		NDT	T‐NMOS + NMOS	3	0–50 V (15–30 V)	PVCR 10^3^		
	n‐MoS_2_/p‐WSe_2_	AAT	AA only	N/A	−40 to 0 V	PVCR 200	2016	^[^ ^95^ ^]^
	WSe_2_/SnSe_2_	NDR (ED)	NDR only	N/A	1–3 V	PVCR 4	2019	^[^ ^96^ ^]^
	BP/SnSe_2_	NDR (ED)	NDR only	N/A	−0.1 to 0.5 V (0–0.3 V)	PVCR 1.8	2015	^[^ ^97^ ^]^
	BP/ReS_2_	NDR (ED)	NDR + PMOS	3	0–25 V (12–18 V)	PVCR 4.2	2016	^[^ ^52^ ^]^
	BP/HfS_2_	NDR (ED)	SRAM	3	0–1.7 V (0.7, 1.1, 1.5 V)	PVCR 2.3	2020	^[^ ^98^ ^]^
	HfS_2_/pentacene	NDR (ED)	Amplifier	3	N/A	PVCR 1.64	2020	^[^ ^99^ ^]^
Organic heterojunction	*α*‐6T/PTCDI‐C8	AAT (PN)	T‐NMOS + PMOS	3	0–30 V (17–19 V)	PVCR 100–10 000	2018	^[^ ^101^ ^]^
	DNTT/PTCDI‐C13	NDT (PN)	T‐PMOS+ NMOS	3	0–50 V (14–20 V)	N/A	2019	^[^ ^103^ ^]^
	P(NDI2OD–Se2)/P(DPP2DT‐T2)	NDR (PN)	SRAM	3	0–40 V (0, 13, 29 V)	PVCR 100	2020	^[^ ^74^ ^]^
		NDT	N/A	N/A	−100 to 100 V	PVCR 13000		
Graphene homojunction	Graphene/Pt(Al) Strip	NDT	T‐CMOS	3	0–2 V (0.6–1.4 V)	N/A	2016	^[^ ^54^ ^]^
	Graphene/ R6G	NDT	T‐PMOS + NMOS	3	−40 to 40 V (−10 to 20 V)	N/A	2018	^[^ ^105^ ^]^
QDs	ZnO,	T‐NMOS	T‐NMOS + NMOS	>3	0–5 V	N/A	2019	^[^ ^34^ ^]^
	SiO*_x_*,	T‐NMOS	T‐NMOS + NMOS	3	0–2 V	N/A	2015	^[^ ^16^ ^]^
	GeO*_x_*	T‐NMOS	T‐NMOS + NMOS	3	0–5 V	N/A	2011	^[^ ^15^ ^]^
Memristor	Pt/ZnO/Pt	N/A	N/A	3	−2 to 2 V writing	27 states	2019	^[^ ^107^ ^]^
	Pt/HfO*_x_*/ITO	N/A	N/A	3	−2 to 2 V writing	32 states	2020	^[^ ^106^ ^]^
Spintronics	III–V	N/A	N/A	N/A	N/A	N/A	2010	^[^ ^108^ ^]^
Other	Si	CMOS	T‐CMOS	3	0–2 V	N/A	2019	^[^ ^53^ ^]^

^a)^ Types of target devices. Detailed criteria are provided in parentheses, where applicable. RTD: resonant tunneling diode; ED: Esaki diode; PN: p‐type/n‐type junction device;

^b)^ Configurations are set corresponding to equivalent circuits in Figure 1, where applicable;

^c)^ Voltage differences between highest and lowest logic states. The range for the intermediate state is included in parentheses;

^d)^ FOM refers to major figure of merits for the component device, which includes peak‐to‐valley current ratio (PVCR) of NDR/NDT devices at room temperature.

### 2.2 Emerging Materials for Multivalued Logic Gates

**Figure 3:**
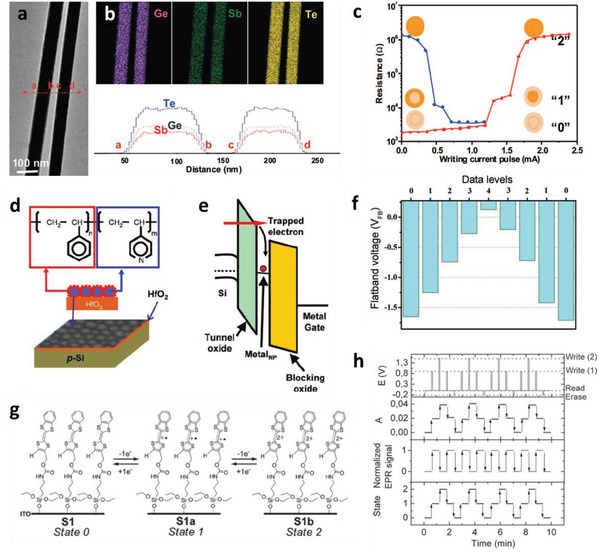
a) Transmission electron microscopy (TEM) image of two Ge_2_Sb_2_Te_5_ nanowires, and b) their elemental analysis (top) and spatial distributions of constituent elements (bottom). c) Variation in resistance with writing current pulse. a–c) Reproduced with permission.^[^ ^6^ ^]^ Copyright 2008, American Chemical Society. d) Schematic of metal‐nanoparticle‐based MVL device and e) corresponding energy‐band diagram of memory device incorporated with charge‐trapping elements. f) Multilevel memory behavior in nanoparticle‐based device. d–f) Reproduced with permission.^[^ ^5^ ^]^ Copyright 2011, Wiley. g) Schematic of electrochemically multistable molecule layer. h) Multivalued nonvolatile switching behavior. g,h) Reproduced with permission.^[^ ^109^ ^]^ Copyright 2011, American Chemical Society.

New material systems such as inorganic heterostructure nanowires, metal nanoparticles, polymers, and organic molecules have been introduced for increasing the information density in a unit device.^[^ ^6^, ^7^, ^55^, ^56^ ^]^ The corresponding properties of the emerging materials, e.g., phase transition, charge trapping, and reversible redox, have been used to demonstrate their multivalued electrical characteristics. Phase‐transition‐based memory switching behavior was first studied since the discovery of semiconducting properties of chalcogenide glass in the mid‐1950s. A device that exploits this unique behavior of chalcogenide glass is termed phase‐change random access memory (PRAM). Subsequently, fast and reversible switching and memory effects were reported in the Si–Te–As–Ge (STAG) system. After a decade, the most commonly used material system was a Ge–Sb–Te (GST) ternary alloy, which showed much faster phase transition (i.e., recrystallization) than the STAG system.^[^ ^57^ ^]^ Later, the GST chalcogen alloy was nanostructured into 1D core/shell heterostructure nanowires, which were used in multistate memory applications.^[^ ^6^ ^]^ The heterostructure nanowire consists of a Ge_2_Sb_2_Te_5_ core and GeTe shell, where each of these chalcogenide alloys shows a reversible amorphous‐to‐crystalline phase transition under electric‐field‐induced Joule heating. As the applied voltage increases, the core and shell alloys subsequently undergo phase transitions at different voltages and two separate threshold switching voltages are observed. These subsequent and reversible crystallinity transitions result in distinct electronic states, which are applicable to multilevel logic circuits. **Figure** **3**a shows a transmission electron microscopy (TEM) image of Ge_2_Sb_2_Te_5_ nanowires and results of their elemental mapping, which reveal the spatial distributions of the Ge, Sb, and Te elements (Figure 3b). The electrical resistance of the Ge_2_Sb_2_Te_5_/GeTe heterostructure nanowires has three distinct states (low, intermediate, and high, defined as “0,” “1,” and “2,” respectively) under variation of the pulse duration from 100 to 300 ns, which causes an amorphous‐to‐crystalline phase transition (Figure 3c). This phase‐transition‐based approach can be further tuned by structural or chemical modulations. However, scalable synthesis of heterostructure nanowires in large quantities and their precise positioning or assembly are limitations to their practical application to devices.^[^ ^19^ ^]^

Another approach to storing information at multiple levels is based on metal‐particle‐array‐induced controlled capacitive coupling of trapped electrons.^[^ ^5^, ^58^ ^]^ This concept was first applied to floating‐gate‐assisted nonvolatile memory devices for prolonging the discharge time of the stored information in an effort to overcome the limitation of conventional flash memory devices.^[^ ^59^, ^60^, ^61^, ^62^, ^63^ ^]^ Metal nanoparticles have emerged as an effective material for storing information in discrete charge‐trapping sites by this approach to achieve improved reliability of memory devices. Multilevel information storage under the condition of identical device geometry has been achieved using well‐defined metal nanoparticle arrays as gate‐voltage‐controllable charge‐trapping sites. For example, a gold‐nanoparticle‐embedded HfO_2_ layer is used as the gate dielectric to generate gate‐voltage‐controllable multilevel memory states; here, the gold nanoparticles function as the charge‐trapping elements (Figure 3d). The highly ordered arrays of gold nanoparticles as a charge‐trapping layer generate saturated flat‐band voltages for programming and erasing of memory devices (Figure 3e).^[^ ^5^ ^]^ Figure 3f shows five‐level information storage in the gold‐nanoparticle‐assisted charge‐trapping memory devices. In this work, multilevel information storage was achieved using metal nanoparticles as the charge‐trapping elements to demonstrate the saturation of the programming and erasing states. Nanoparticle synthesis at low temperatures can be an additional advantage in their potential applications in the field of flexible electronics. Multilevel information storage characteristics have also been achieved by the immobilization of multiple redox‐active molecules that can be reversibly oxidized or reduced at certain potentials, which enables information storage in discrete redox states.^[^ ^7^ ^]^

Surface‐confined molecular‐charge‐storage devices capable of multilevel information storage in a unit device have also been demonstrated. For example, some organic electroactive molecules such as tetrathiafulvalenes (TTFs) show multiple redox states and a self‐assembled monolayer of a TTF derivative exhibits outstanding switching behaviors.^[^ ^7^, ^109^ ^]^ As shown in Figure 3g, a sequence of voltage pulses generates S1, S1a, and S1b—defined as state 0, state 1, and state 2, respectively—of the TTF monolayer structure. Figure 3h shows the corresponding three‐state nonvolatile switching behavior of the TTF‐monolayer‐based device. Another example is an osmium polypyridyl complex–based self‐propagating molecular assembly, which is a bistable (“0” or “1” when fully reduced to Os^2+^ or fully oxidized to Os^3+^, respectively) electrochromic metalorganic functionalized film. Such chemical reactions in molecule‐enabled multilevel information storage can be another novel route to realizing memory devices in the future.^[^ ^7^ ^]^

### 2.3 Negative Differential Resistance and Negative Transconductance

Physical manifestation of the electrical intermediate state in emerging materials has proved to be a promising candidate approach for the realization of multistate devices for MVL gates. A more classic approach to MVL operation is based on the nonlinear and nonmonotonic electrical behaviors of NDR and NTC. Since Leo Esaki's first demonstration of NDR behavior in heavily doped germanium tunnel junctions in 1958,^[^ ^64^ ^]^ the feasibility of exploiting the nonlinearity of electrical behavior has motivated intensive research for the development of new types of electronic device designs. However, the complexity of fabrication of such tunneling devices and a dearth of suitable candidate materials have severely limited the practical application of Esaki diodes in the emerging era of the CMOS technology. Various other devices have been proposed for inducing NDR behavior using different operating principles, including resonant tunneling diodes (double‐barrier quantum well devices),^[^ ^50^, ^51^, ^65^ ^]^ Gunn diodes (transferred‐electron devices),^[^ ^66^ ^]^ impact‐ionization avalanche transit time (IMPATT) diodes,^[^ ^67^, ^68^ ^]^ single‐electron transistors,^[^ ^69^, ^70^ ^]^ and molecular‐junction devices.^[^ ^71^, ^72^, ^73^ ^]^ In addition to two‐terminal devices, the three‐terminal transistor architecture has also been widely investigated as an alternative nonlinear electronic component for inducing NTC behavior; the PVCR of three‐terminal transistor devices can be improved considerably, i.e., by 2–3 orders of magnitude, compared with that of two‐terminal devices.^[^ ^74^ ^]^ Because of such persistent research efforts, the advanced NDR and NTC technologies have found applications in the field of electronics and been widely used in direct current (DC) amplifiers and unretarded terahertz electronic oscillators.

In the 2000s, as advancements in computing technology gradually reached their limits, finding an alternative to the conventional CMOS‐based binary logic system became crucial. This technological need highlighted another important aspect of NDR devices: the innate multistable operation capability of NDR and NDT devices with prospects for realizing higher‐radix (>2) logic operations. Logic gates containing NDR devices were also acknowledged for their distinct advantages of low power consumption with self‐latching in their bistable states, high noise tolerance, and high compactness in circuit pipelining.^[^ ^49^ ^]^ Furthermore, evolution of materials based on atomically thin 2D semiconductors (Section 2.3.1 and 2.3.3) and chemically versatile organic semiconductors (Section 2.3.2) opened up new avenues for practical and reliable fabrication of NDR and NTC devices. In the following subsection, we summarize the evolution of materials used to realize NDR and NTC devices and their corresponding operating principles.

#### 2.3.1 2D van der Waals Heterostructures

The recent evolution of materials has enabled large‐area and high‐throughput synthesis of various atomically thin 2D materials such as semiconducting TMDCs, BP, graphene, and insulating hexagonal boron nitride (h‐BN). The possibility of facile formation of mechanically, electrically, and energetically sharp interfaces in vertically stacked heterostructures of these 2D materials bound through van der Waals (vdW) interactions has been demonstrated.^[^ ^75^, ^76^, ^77^, ^78^, ^79^, ^80^ ^]^ The high sharpness of the interfaces in such vdW heterostructures offers distinct advantages in the realization of high‐quality heterojunctions for tunneling compared with conventional crystalline Si, Si–Ge,^[^ ^65^, ^73^, ^81^, ^82^, ^83^, ^84^ ^]^ and III–V semiconductor heterojunctions. Tunneling junctions made of such conventional bulk materials are largely limited by predominant lattice mismatches, dislocations, surficial imperfections, and dangling bonds; their production costs are therefore high and their tunneling behaviors are unstable.

**Figure 4:**
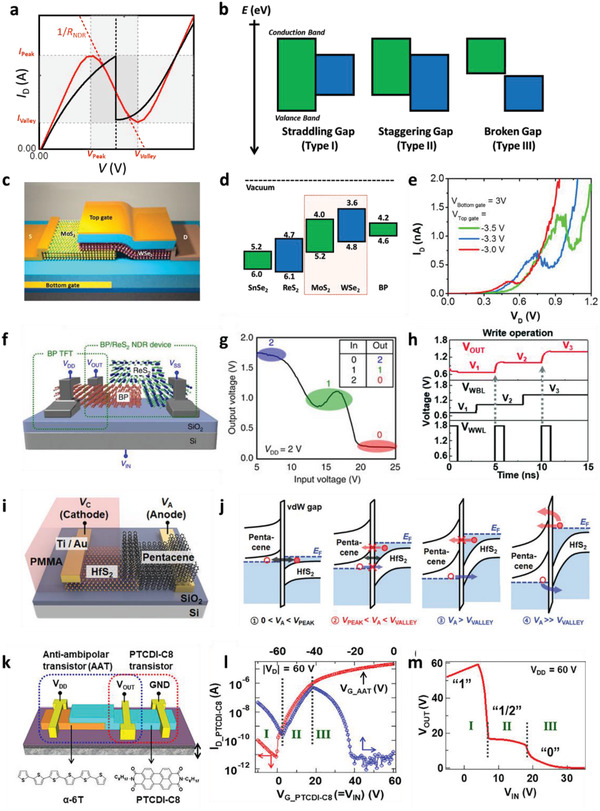
a) Typical current–voltage characteristics of NDR device and NTC device. b) Schematic illustration of various types of band alignments. c) Structure, d) energy‐level diagram, and e) NDR characteristics of MoS_2_/WSe_2_ heterojunction device. c,e) Reproduced with permission.^[^ ^93^ ^]^ Copyright 2015, American Chemical Society. d) The energy levels (SnSe_2_,^[^ ^96^ ^]^ ReSe_2_,^[^ ^52^ ^]^ MoS_2_,^[^ ^94^ ^]^ WSe_2_,^[^ ^96^ ^]^ and BP^[^ ^52^ ^]^) are adopted from the literature. f) Structure, g) STI operation, and h) SRAM operation of BP/ReS_2_ heterojunction device. f,g) Reproduced with permission.^[^ ^52^ ^]^ Copyright 2016, Springer Nature. h) Reproduced with permission. Copyright 2020, Royal Society of Chemistry.^[^ ^98^ ^]^ i) Structure and j) operating principle of pentacene/HfS_2_ heterojunction device.^[^ ^99^ ^]^ k) STI device structure, l) transfer characteristics, and m) STI operation of *α*‐6T/PTCDI‐C8‐based AAT circuit. k–m) Reproduced with permission.^[^ ^101^ ^]^ Copyright 2018, American Chemical Society.

Importantly, the heterojunction quality determines the reliability of band‐to‐band tunneling (BTBT) behaviors, which govern the nonlinearity of electrical characteristics.^[^ ^85^ ^]^ Furthermore, through engineering of the band alignment at heterojunctions—categorized as type‐I (straddling gap), type‐II (staggered gap), and type‐III (broken gap) alignments, the PVCR as well as the fold‐back feature of the current–voltage characteristics of an NDR device (**Figure** **4**a,b) can be controlled and optimized as required. For example, the dual‐gated transistor architecture based on the multistacked graphene/h‐BN/graphene vdW heterostructure exhibits field‐effect tunneling diode characteristics because of the explicit control of the top and bottom graphene layers by the dual‐gate structure.^[^ ^79^, ^86^, ^87^, ^88^, ^89^ ^]^ Under application of a sufficiently large electric field at low temperature, the vdW stacked heterostructure can exhibit NDR characteristics. Further progress has been made using bilayer graphene in the graphene/h‐BN/graphene structure. The rotationally aligned bilayers enable momentum‐conserving resonant tunneling through the h‐BN layer, and this, in turn, induces noticeable NDR behavior at room temperature, with a PVCR of up to 4.^[^ ^90^ ^]^ The fold‐back feature, peak voltage, and PVCR of an NDR device can be controlled easily by the application of a gate voltage when the device is fabricated in the three‐terminal transistor architecture. Similarly, the multiple vdW‐stacked MoS_2_ layers also exhibit resonant‐tunneling based NDR behaviors.^[^ ^91^ ^]^

Following confirmation of the versatility of tunneling of the vdW heterostructure, the type‐II band alignment between vertically stacked MoS_2_ and WSe_2_ has been widely investigated because the strong coupling between the layers enables efficient modulation of the BTBT behaviors. On top of the epitaxial graphene, Lin et al. has demonstrated that the sequential growth of WSe_2_ and MoS_2_ (or MoSe_2_/WSe_2_) can be utilized to fabricate a vertical vdW heterojunction. Similarly to the graphene/h‐BN/graphene vdW heterostructure, the atomically stitched MoS_2_/WSe_2_ vdW heterojunction resulted in a sharp NDR behavior from the resonant tunneling between layers.^[^ ^92^ ^]^ To further apply this structure in the electronic device, Roy et al. showed that, in a vdW heterostructure including the MoS_2_ and WSe_2_ layers, the charge densities and electrical potentials of the layers can also be explicitly controlled by employing a dual‐gate structure.^[^ ^93^ ^]^ Electrostatic doping of each layer to different extents strengthens or weakens the BTBT, which enables control of the NDR behavior of the heterostructure. Figure 4c,d shows the device architecture and the corresponding energy‐level diagram, respectively. The dependence of the NDR behavior on the top‐gate and bottom‐gate voltages promisingly demonstrates that the NDR characteristics (Figure 4e), represented by the PVCR and the peak voltage, can be controlled easily in this architecture. As described in an earlier section, these modulations constitute the basis for the ideal operation of MVL gates, which is evaluated in terms of the stability and equiprobable accessibility of the intermediate states. However, complex fabrication processes are required for the double‐gate structure, which limits the applicability of this approach to MVL gates. In view of this limitation, Nourbakhsh et al. further investigated the electrical characteristics of MoS_2_/WSe_2_ vdW heterostructures and showed that the fine tuning of the thicknesses of both the TMDC layers can induce both NDR and NTC behaviors in a single‐gate architecture even at room temperature.^[^ ^94^ ^]^ By using WSe_2_ as the channel layer of the PMOS transistor and the MoS_2_/WSe_2_ heterostructure as the NTC device in the CMOS architecture (Figure 2e), they succeeded in achieving STI operation with high noise margins for the intermediate states. However, this MoS_2_/WSe_2_ heterostructure was also capable of showing antiambipolar behavior. Li et al. showed that the vdW heterostructure can show not only tunneling‐based NDR behavior but also charge‐transport‐mediated transfer characteristics in the form of a peak function.^[^ ^95^ ^]^ The detailed operating principles and evolution of materials with antiambipolar properties are discussed in a later section.

Fan et al. showed that a combination of WSe_2_ and another TMDC, SnSe_2_—which has the broken‐gap alignment (type‐III) by default—could also be used to induce NDR behavior.^[^ ^96^ ^]^ Precise modulation of the BTBT behavior could be achieved in the structure with this band alignment, which manifested as a high PVCR exceeding 4. They further demonstrated the tunability of the NDR behavior via electrostatic doping of the TMDCs under application of a back‐gate biases well as via introduction of a thickness‐controlled tunneling barrier, h‐BN, between the WSe_2_ and SnSe_2_ layers. The tunneling barrier was introduced to demonstrate the adverse effects of SnSe_2_ oxidation on BTBT behaviors, but this introduction could also be a possible approach to optimizing the NDR behavior. .

On the other hand, earlier efforts to find suitable candidate materials having the broken‐gap (type‐III) alignment also revealed that a BP/SnSe_2_ heterostructure can exhibit NDR behavior even at room temperature.^[^ ^97^ ^]^ An Esaki diode based on the BP/SnSe_2_ heterostructure showed a PVCR of 2 at 300 K, which was also highly dependent on the oxidation of SnSe_2_. The search for more reliable and stable 2D materials yielded a BP‐based heterostructure in which the SnSe_2_ was replaced with ReS_2_.^[^ ^52^ ^]^ Figure 4f shows a schematic diagram of the STI architecture based on the BP/ReS_2_ heterostructure. The BP functions as the channel layer of the PMOS component in the NDR + PMOS architecture (Figure 2f), whereas the BP/ReS_2_ vdW heterostructure functions as the two‐terminal NDR device in this architecture. With an increase in the gate voltage, the resistivity of the PMOS component changes and the peak current of the NDR component varies marginally. As a result, the STI shows stable intermediate operating points in the NDR region of the electrical characteristics of the BP/ReS_2_ device (Figure 4g). In this vein, Kim et al. further extended the application of the BP/ReS_2_ heterostructure to ternary static random access memory (T‐SRAM) by incorporating BP/HfS_2_ as the secondary NDR component.^[^ ^98^ ^]^ The double‐valley structure in this NDR device provided three stable operating points, which could stably store the charges in three states. Figure 4h shows the write operation of the T‐SRAM that resulted in three stable output states in the latch operation.

#### 2.3.2 Organic Semiconductor Heterojunctions

Following the successful realization of NDR, NTC, and MVL gates using 2D materials, the incorporation of organic semiconductors into such configurations opened up avenues for further advancements in the field of MVL devices. The chemical versatility of organic semiconductors enables fine tuning of their electrical and energetic properties, and they can thus satisfy the material criteria extracted from the above discussions. Very recently, Jung et al. reported a tunneling diode based on a pentacene/HfS_2_ heterojunction.^[^ ^99^ ^]^ The tunneling diode exhibited NDR properties that could be controlled by geometrical modulation of the pentacene layer (Figure 4i). The accumulated understanding of the pentacene thin film growth in previous field‐effect‐transistor researches enabled almost defectless growth of them on the HfS_2_ surface, which yielded a high‐quality interface with the HfS_2_ layer similar to the interfaces in the 2D vdW heterostructures composed of TMDCs. The broken‐gap (type‐III) alignment of the pentacene/HfS_2_ heterostructure facilitated tunneling‐derived NDR behavior, and the device showed a PVCR of up to 1.64 at room temperature. They further demonstrated that the incorporation of double anodes with different distances to the cathode (i.e., two different channel lengths) in a single‐device architecture led to double NDR behavior.

Organic materials have also been used to induce NTC behavior in an all‐organic heterojunction transistor architecture. In this architecture, laterally deposited p‐type and n‐type semiconductors form a heterojunction at the center of the channel region, and consequently, the imbalance of the charge transport properties under gate bias (transconductance) can lead to nonlinear electrical behavior. A typical representation of these behaviors is antiambipolar charge transport, wherein the transconductance resembles a peak function under a gate voltage. Kobashi et al. demonstrated that a transistor with *α*‐sexithiophene (*α*‐6T) as the p‐type channel and *N*,*N*′‐dioctyl‐3,4,9,10‐perylenedicarboximide (PTCDI‐C8) as the n‐type channel in the heterojunction transistor (Figure 4k). The device architecture exhibited an antiambipolar behavior with a PVCR (or on/off current ratio) of up to 10^2^ for p‐type operations and 10^4^ for n‐type operations.^[^ ^100^, ^101^ ^]^ They further demonstrated that both the peak voltage and the PVCR could be tuned by changing the *α*‐6T channel thickness. They then utilized this excellent PVCR and tunability of the transconductance of the demonstrated heterojunction transistor to synthesize an STI (Figure 4l,m).^[^ ^102^ ^]^ Utilization of the unstacked region of PTCDI‐C8 as the channel of the NMOS transistor in the T‐CMOS configuration (Figure 2e) enabled this simple device architecture to have a stable intermediate state with accessibility almost equiprobable to that of the high and low states of the ternary inverter.

However, in an AAT‐based STI, an issue rises where the AAT should exhibit nonideal asymmetric electrical characteristics with a higher off‐state current level than that of the load transistor ensure a clear transition between high (“2”) and low (“0”) logic states (Figure 4l). Yoo et al. proposed a modification to the heterojunction transistor architecture as a plausible approach to achieving this condition.^[^ ^103^ ^]^ They used the p‐type dinaphtho [2,3‐b:2′,3′‐f] thieno[3,2‐b] thiophene (DNTT) and n‐type *N*,*N*′‐ditridecylperylenediimide (PTCDI‐C13) in the transistor architecture. Instead of using the conventional structure of separate n‐type and p‐type channel regions with a partial overlap in the middle, they connected both the source electrode and the drain electrode to the p‐type semiconductor and vertically stacked the n‐type semiconductor underneath one‐half of the p‐type layer near the drain electrode. In this architecture, the turn‐on and turn‐off of the device were governed by the p‐channel of DNTT and the partial accumulation of charges in PTCDI‐C13 in a certain voltage range produced the NTC behavior. They used the unstacked region of PTCDI‐C13 as the channel of the NMOS transistor in the T‐CMOS configuration (Figure 2e) to realize the inverter function. The clear inversion of the on‐state and off‐state between the NMOS and the NTC‐PMOS transistors resulted in high signal swing with wide noise margins for the intermediate states.

Despite the possible undesirable increase in circuit complexity, an architecture with a simple electrical connection of p‐type and n‐type organic FETs can also be used to produce NDR and NDT behaviors. Jeon et al. fabricated a series‐connected array of n‐type and p‐type ambipolar transistors, poly(4‐([2,2′‐biselenophen]‐5‐yl)‐2,7‐bis(2‐octyldodecyl)‐3a1,5a1‐dihydrobenzo[lmn][3,8]phenanthroline‐1,3,6,8 (2H,7H)‐tetraone) (P(NDI2OD‐Se2)) and poly(3‐([2,2′:5′,2″‐terthiophen]‐5‐yl)‐2,5‐bis(2‐decyltetradecyl)‐6‐(thiophen‐2‐yl)‐2,5‐dihydropyrrolo[3,4‐c]pyrrole‐1,4‐dione) (P(DPP2DT‐T2)), respectively.^[^ ^74^ ^]^ Because of the multithreshold voltage of the circuit, the transfer curve of the circuit showed a W‐shaped double fold‐back NTC behavior with a very high PVCR exceeding 10^4^. Furthermore, they tuned the electrical characteristics of the two organic semiconductors by using polymethylmethacrylate (PMMA):poly(vinylidenefluoride‐*co*‐trifluoroethylene) (P(VDF‐TrFE)) as the gate dielectric. Wide tunability ranges of both the peak voltage and the PVCR depending on different extents of charge‐transfer doping were observed. Furthermore, precise modulation of the gate voltage, drain voltage, and doping amount yielded NDR behavior with the highest reported PVCR value of over 10^2^. Jeon et al. then successfully demonstrated ternary latch operations using this NDR device.

#### 2.3.3 Graphene Homojunctions

**Figure 5:**
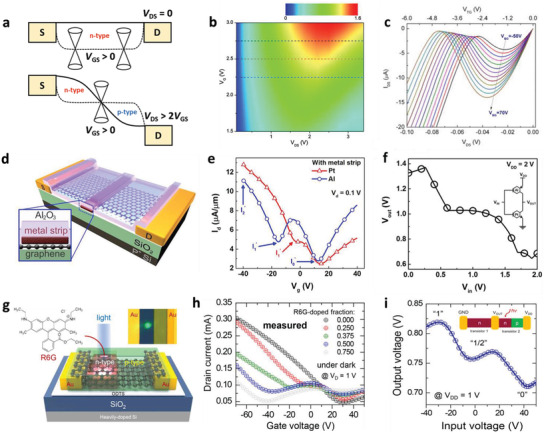
a) Schematic energy‐level diagrams of p/n homojunction formed in graphene by voltage application. b) Contour map and c) current–voltage plot for NDR transfer characteristics as functions of applied biases. a,b) Reproduced with permission.^[^ ^110^ ^]^ Copyright 2012, American Chemical Society. c) Reproduced with permission.^[^ ^89^ ^]^ Copyright 2013, AIP Publishing. d) Device structure, e) NTC transfer characteristics, and f) STI operation of graphene transistor doped with thin metal strip. d–f) Reproduced with permission.^[^ ^54^ ^]^ Copyright 2016, Springer Nature. g) Schematic illustration of operation of graphene transistor doped with R6G organic dye under light illumination, h) NTC transfer characteristics of device, and i) STI operation of device. g–i) Reproduced with permission.^[^ ^105^ ^]^ Copyright 2018, American Chemical Society.

As described in Section 2.3.1, the field‐effect tunneling and resonant tunneling behaviors of a graphene‐containing vdW heterostructure have been shown to be promising for producing nonlinear electrical behavior. Apart from the tunneling behavior, graphene itself can also be used to produce nonlinear electrical behavior because of its unique Dirac‐cone band structure. The gapless symmetric band structure of graphene is conducive for direct charge‐carrier doping via application of electrical potential. A slight change in the Fermi level above or below the Dirac point results in a complete inversion of the charge carrier type as well as a gradual shift in the minimum conductivity voltage in the transfer curves of the graphene transistor.^[^ ^104^ ^]^ Similarly, one can imagine that in a three‐terminal transistor architecture, a homojunction can be formed within the channel region of the transistor by the application of suitable gate and drain voltages such that both can cause similar charge‐carrier doping of graphene (**Figure** **5**a). Wu et al. suggested that this homojunction can act as the p/n heterojunction in heterojunction transistors.^[^ ^110^ ^]^ Figure 5b shows the contour map of the transfer curves along with the drain voltage (*x*‐axis) and gate voltage (*y*‐axis). These curves reveal a clear and smooth transition from the positive differential resistance (PDR) to the NDR in a certain range of the gate voltage, which intensifies as the gate voltage increases. Liu et al., further demonstrated the possibility of realizing various non‐Boolean logic circuits including NOT (inverter), NAND and XOR gates through the fine adjustments of the applied gate and drain voltages in a circuit. (Figure 5c) They further showed that the NDR behavior appeared not only in drift‐diffusion regime but also in ballistic regime, appearing as the intrinsic property of graphene.^[^ ^89^ ^]^ Sharma et al. also suggested that when a dual‐gate structure is used to control the total channel series resistance, the competition between the drift velocity and the charge carrier density can lead to NDR behavior.^[^ ^88^ ^]^

Kim et al. devised a more practical strategy for achieving nonlinear electrical characteristics.^[^ ^54^ ^]^ They deposited a thin strip of Al or Pt directly on top of the graphene surface at the center of the channel region as shown in Figure 5d. The resultant spatially different doping characteristics induced by the Al strip resulted in a p/n/p staggered homojunction that was characterized by multiple Dirac voltages in the transfer characteristics, as shown in Figure 5e. They also found that low‐temperature, high‐pressure hydrogen annealing of the graphene transistor with the Pt strip increased the work function of Pt to beyond that of intrinsic graphene, which enabled the formation of a reverse n/p/n homojunction in this transistor. These VTC behaviors were then utilized to realize STI operation of the Pt‐deposited and Al‐deposited graphene transistors in the T‐CMOS configuration (Figure 5f). The operation mechanism of these devices was similar to the STI operation mechanism shown in Figure 2d,e. Kim et al. further expanded this spatial doping concept.^[^ ^105^ ^]^ They used an organic dye (rhodamine 6G (R6G)) to simulate the partial p‐doping condition of graphene as shown in the schematic device architecture in Figure 5g. The NTC behavior, including the peak voltage, current level, and fold‐back structure, could be controlled easily by tuning the width of the R6G strip covering the graphene surface (Figure 5h). Finally, by using the part of the graphene surface that was not covered by R6G as the channel of the n‐type load transistor, they again realized STI operation according to the mechanism shown in Figure 2e (Figure 5i). Furthermore, by using the photo‐induced doping characteristics of R6G dyes, the graphene homojunction could be finely controlled so that the STI circuit further demonstrated its capability in the photonic applications.

#### 2.3.4 Prospective Approaches for NDR‐Based MVL Gates

We have thus far summarized recent efforts targeted at inducing NDR and NTC behaviors in terms of the evolution of materials useful for this purpose and the architectural designs of the corresponding devices. The main research trend has been centered on the utilization of controlled‐tunneling behaviors as well as lateral p/n junction configurations. The most recent advances in these architectures have demonstrated that the nonlinearity in the electrical characteristics is characterized by the PVCR within the range of 10. The bare minimum value of the PVCR for the logic operation is about 2; however, depending on the target practical application, a value greater than 10^4^ would be required for stable operation.^[^ ^100^ ^]^ This highlights the need for further advancements in both materials and device architectures. Fortunately, multitudes of options remain to be explored. We pointed out earlier that various devices besides the Esaki diode have been developed to produce NDR behavior, such as resonant tunneling diodes (double‐barrier quantum well devices), Gunn diodes (transferred‐electron devices), IMPATT diodes, single‐electron transistors, and molecular‐junction devices. For example, resonant tunneling diodes with In_0.52_A1_0.48_As as the central barrier layer sandwiched between two In_0.53_Ga_0.47_As quantum wells have successfully shown a PVCR of up to 144 at room temperature, whereas the PVCR of a recently reported Esaki diode based on an inorganic GaSb–InAsSb structure remains under 10, as is typically the case with vdW‐heterostructure‐based nonlinear devices. Devices based on mechanisms completely different from tunneling or heterojunctions can also be explored, including IMPATT diodes and Gunn diodes. Recently developed semiconducting materials as well as their precise deposition, alignment, and stacking methodologies can be employed in avalanche current generation and subsequent control of the transit time delay, as is done in IMPATT diodes. The application of these materials can be further expanded to variants of IMPATT didoes, such as barrier injection and transit time (BARITT) diodes and quantum‐well injection transit time (QWITT) diodes. Various emerging materials other than III–V semiconductors (e.g., GaAs, GaN, and InP), such as monolayer TMDCs and multilayer graphene, can also be applied to Gunn diodes, which utilize the satellite valley in the dispersion relations. Molecular‐junction devices, which are based on a reversible transition between conduction and insulation through a voltage‐derived redox reaction, also have scope to be investigated extensively, as discussed in Section 2.2. Moreover, single‐electron transistors based on tunneling through isolated single‐electron islands with discrete energy levels have promising prospects as hinted by the recent rapid advancements in QD‐based technology.

### 2.4 Quantum‐Dot‐Gate Field‐Effect Transistors

In the previous section, we discussed NDR behavior as a candidate mechanism for realizing MVL applications. Although NDR devices have several advantages, e.g., a wide choice of materials, they have not yet been fully explored for direct incorporation into logic circuits because two or more semiconducting materials are required to intentionally adjust the energy bandgap alignment, which highly complicates their fabrication process. Furthermore, tunneling‐based operating mechanisms can generate excessive leakage current and/or valley current, and the working temperature of such devices needs to be low.

QD‐gate field‐effect transistors (QDGFETs) are another class of devices developed in an effort to produce multivalued electrical characteristics.^[^ ^15^, ^16^, ^34^ ^]^ 0D QDs have, in general, received considerable attention as a promising material platform in FET applications because of their advantages such as charge leakage reduction and device stability improvement. More important than the general properties of QDs, their presence plays a key role in the generation of an additional intermediate state as a charge storage element, which enables handling of a greater density of information in a given device dimension.

**Figure 6:**
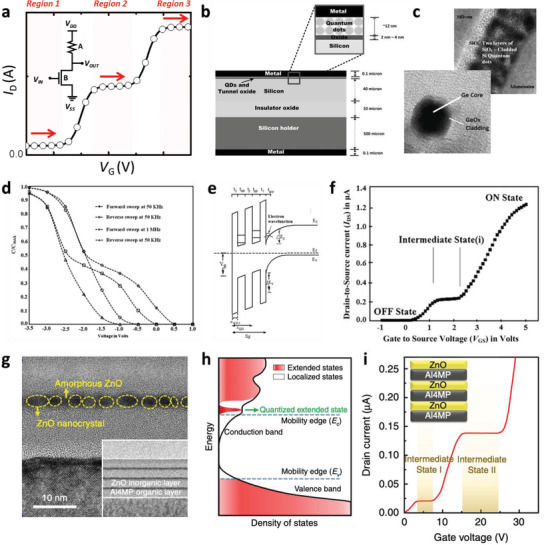
a) Schematic of three‐level transfer curve; the inset shows the circuit symbol of the T‐NMOS + R configuration. b) Device geometry of QDGFET on SOI substrate. c) TEM images of SiO*_x_*‐QD‐embedded and GeO*_x_*‐QD‐embedded structures. d) Multivalued capacitance–voltage characteristics of QDGFETs. f) Multivalued transfer characteristics of a QDGFET showing an intermediate state between the conventional on‐state and off‐state. b–f) Reproduced with permission.^[^ ^15^, ^16^ ^]^ Copyright 2011, 2015, The Institution of Engineering and Technology. g) TEM image of amorphous ZnO hybrid layer embedded with ZnO nanocrystals. h) Schematic of total density of states of hybrid nanolayer having a quantized extended state. i) Transfer characteristics of quaternary device with two intermediate states. g–i) Reproduced with permission.^[^ ^34^ ^]^ Copyright 2019, Springer Nature.

When a QDGFET is fabricated by embedding QDs into the dielectric layer of a conventional FET structure, the representative transfer curve of the QDGFET shows an intermediate state (region 2) in addition to the conventional binary on‐state (region 3) and off‐state (region 1), as shown in **Figure** **6**a.^[^ ^15^, ^16^ ^]^ The inset is a circuit diagram of a resistive‐load inverter, which is a commonly used structure for the implementation of QDGFETs, as also detailed in Figure 2. The QDGFET device structure is composed of two layers of QDs deposited on top of the gate insulator (Figure 6b), whereas the rest of the device structure is the same as a conventional FET structure. A cross‐sectional high‐resolution TEM image reveals the presence of two layers of SiO*_x_*‐cladded Si QDs between a SiO_2_ gate insulator and an Al gate electrode (Figure 6c). GeO*_x_*‐cladded Ge QDs have also been used to generate the intermediate state in QDGFETs. These QDs embedded in a gate dielectric layer provide additional charge storage capacity by facilitating carrier tunneling from the inversion channel, which is verified by low‐frequency (50 kHz) capacitance–voltage (*C*–*V*) measurements, as shown in Figure 6d. The energy‐band diagram of a QDGFET device as calculated by solving the Schrödinger and Poisson equations reveals the working principle of generation of the intermediate state (Figure 6e).^[^ ^13^, ^29^ ^]^ From the band structure, the origin of the intermediate state can be described to be carrier tunneling from the inversion channel to QDs present in the gate region. The current flow in the intermediate state is almost independent of the gate voltage because the transconductance (gate voltage–threshold voltage) remains almost the same under application of gate voltage. The transfer characteristics of the QDGFET exhibit three‐state behavior under variation of the applied gate voltage, and the intermediate state is more stable than that in NDR devices, in which case the intermediate‐state current decreases rapidly because of high charge leakage (Figure 6f).^[^ ^13^, ^16^ ^]^ The position of the intermediate state can be controlled even by tuning the structural parameters of the QDs, such as the number of layers, size distribution, and surface coverage uniformity.

Recently, an approach based on ZnO QDs has been used to produce multilevel electrical characteristics.^[^ ^34^ ^]^ In this approach, ZnO QDs are embedded into amorphous ZnO domains that are sandwiched between 4‐mercaptophenol (4MP) molecular layers with Al linkers (Al4MP). A cross‐sectional TEM image shows the ZnO QDs (or nanocrystals) embedded into an amorphous ZnO layer placed between Al4MP organic layers (Figure 6g). This unit composite layer composed of the Al4MP layer and the ZnO‐QD‐embedded ZnO layer can be alternatively stacked as shown in the inset to realize devices beyond the ternary level. As schematically illustrated in Figure 6h, an additional quantized extended state, which originates from the quantum confinement effect induced by the ZnO QDs, is present below the mobility edge of the ZnO layer. This quantized state with a low density of states limits the number of carriers transported and results in stable current saturation to generate the intermediate state. This approach provides, for the first time, quaternary electrical characteristics with an increase in the number of stacked unit layers (Figure 6i). This ZnO‐QD‐based device is implemented into a resistive‐load logic gate and the resultant device successfully demonstrates the feasibility of multilevel circuit operations.^[^ ^30^ ^]^

### 2.5 Other Emerging Approaches

**Figure 7:**
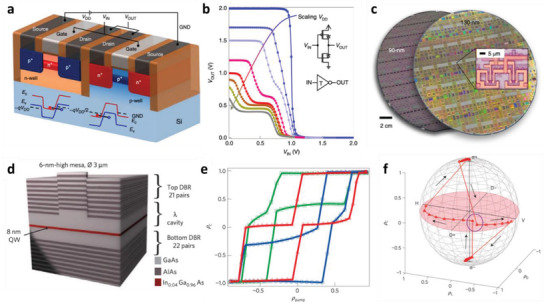
a) Device geometry of BTBT‐based multivalued T‐CMOS transistor. b) Binary‐to‐ternary transition under scaling of applied *V*_DD_ from 2 to 0.7 V. The inset shows a schematic diagram of a T‐CMOS‐based inverter. c) Photograph of 8 in wafers with T‐CMOS device arrays. a–c) Reproduced with permission.^[^ ^53^ ^]^ Copyright 2019, Springer Nature. d) III–V semiconductor‐based nanocavity for polariton confinement. e) Multistability of polaritons. f) Trajectory of pseudospin in Bloch sphere, indicating multistability of polariton spin. d–f) Reproduced with permission.^[^ ^108^ ^]^ Copyright 2010, Springer Nature.

The above‐discussed ternary systems for MVL devices are typically realized via the generation of multithreshold voltage. To overcome the limitations on energy‐efficient and large‐scale fabrication of devices, another configuration, T‐CMOS, has been developed; in this approach, the T‐CMOS transistor generates an intermediate voltage state using a constant off‐state current and it has a single threshold voltage. A quantum‐mechanical BTBT mechanism in a heavily doped p/n junction is used to realize a ternary system based on a constant off‐state current.^[^ ^53^ ^]^ **Figure** **7**a shows a schematic illustration of the T‐CMOS device, and the inset shows the energy‐band diagrams of the BTBT. An off‐state‐induced ternary device is realized by scaling of the constant off‐current down to a sub‐picoampere level via *V*_DD_ scaling at the body‐to‐drain junctions. The *V*_DD_ scaling induces a binary‐to‐ternary transition, as shown in Figure 7b (the inset is a diagram of the T‐CMOS‐based inverter device). This binary (*V*_T_ < *V*_DD_)‐to‐ternary (*V*_T_ > *V*_DD_) transition induced by *V*_DD_ scaling can be a promising mechanism for a novel circuit design, and the practical application of this approach has been demonstrated on an 8 in wafer (Figure 7c).^[^ ^16^ ^]^

Two‐terminal devices with resistive switching capabilities, i.e., memristers, have also been utilized to generate multivalued logic gates. Unlike the two‐terminal NDR devices, the memristers can be programmed to exhibit multiple quantized conductance states with PDRs via an elaborate evolution of conductive filaments under the electrical bias conditions. Xue et al. have demonstrated that the use of a simple Pt/HfO*_x_*/ITO can have stable 32 consecutive quantized states via single‐atom level oxygen manipulation in constructing atomic point contact structures, which allows the implementations of in‐memory ternary logic operations.^[^ ^106^ ^]^ Furthermore, Zhang et al. have implemented 27 univariate ternary logic operations by using a single memristor based Pt/ZnO/Pt structures.^[^ ^107^ ^]^ With proper extraction of the sequential steps for the initialization and writing, the memristor‐based MVL can also be a promising alternative of the three‐terminal multistate device or two‐terminal NDR‐based MVLs. Furthermore, beyond the MVL systems based on volatile physical variables such as voltage‐divided intermediate states, approaches involving memristors can incorporate nonvolatile multilogic states that can function as a viable platform for the non‐von Neumann in‐memory computing.

In addition to the above‐discussed electronic‐device‐based approaches, spintronics‐based approaches have also been developed for the realization of MVL gates.^[^ ^108^ ^]^ In one such spintronics‐device‐based approach, multilevel states were defined using trap‐induced multistability of spin states of microcavity polaritons. Spin multistability refers to the possibility of three or more stable spin states under an excitation condition. A unique semiconductor‐based microcavity structure was designed to achieve spin multistability, in which polaritons could be localized in cylindrical mesa structures as shown in Figure 7d. Spin multistability was achieved using the device geometry as shown in Figure 7e; here, asymmetric multistability was achieved by linear polarization splitting. This complete polariton spin state was further characterized via measurements of the three Stokes parameters and tracking of the trajectory of the pseudospin vector in the Bloch sphere (Figure 7f). This spintronics‐based approach enabled multivalued switching of an ensemble of spins in the solid state.^[^ ^108^ ^]^

## 3 Conclusion and Future Outlook

The rapid progress in research on MVL gates signifies an imminent paradigm shift in the field of electronics toward a new era of hyper Moore's law. The emergence of various new materials, ranging from 0D QDs to vertically stacked vdW heterostructures, has boosted the evolution of MVL technology in recent years. They provide controllable volatile and nonvolatile physical variables for the manifestation of the intermediate states, which can be used to mathematically increase the information density for both von Neumann and in‐memory computing architectures. In this review, we reviewed the current status of research on MVL gates and elucidated the major research trends. Evaluation of the general FOMs of a representative MVL gate, the STI, revealed the design criteria that component devices need to meet in order to realize ideal high‐radix operations. The emerging materials for MVL devices were categorized according to the operating principle of each component device. The evolution of each category of materials was summarized, which provided pointers for future research on MVL gates.

Classically, power dissipation, operating speed, VLSI compatibility, integration level, and circuit complexity are the general FOMs of a logic circuit. The multivalued logic systems fundamentally possess a mathematical superiority in the required level of integration for a given operation than conventional binary logic systems. This advantage can also be beneficious to the power dissipation. However, current Si‐based CMOS technology for binary logic systems is still far superior in all the pre‐existing FOMs due to the difference in the maturity of the technology. Especially, Si‐based materials have distinct advantages in the electrical charge mobility (>10^3^ cm^2^ V^−1^ s^−1^) and susceptibility to the lithographic processing, which currently can compensate limits of the lower radix operations. In this sense, three‐state component devices such as T‐PMOS, T‐NMOS, NDR, NDT, and AAT devices have been extensively investigated for achieving optimal FOMs simultaneously so that they can fully harness the mathematical advantage of MVLs. Furthermore, because of the presence of unique “intermediate” states in MVL gates, they have additional FOMs: 1) equiprobable accessibility to all logic states (i.e., manageable input voltage ranges); 2) a distinctive manifestation of the intermediate state with high noise margin (i.e., small fluctuations in the output voltages and equal distances among them); and 3) operational, physical, chemical, and environmental stability of the intermediate state. These requirements for the intermediate states are not exclusive to the ternary logic systems (radix of 3) and can be equally applied to higher‐radix systems that incorporates more than 1 intermediate state. More complex logic gates such as NAND and NOR universal gates can also be synthesized based on the multistate component devices, which shares the same FOMs and thus can be optimized with the same philosophy. To this end, various approaches based on emerging materials have recently been developed to meet these new criteria. However, despite the rapid progress in the field of MVL gates, the current research is still in the nascent stage in terms of realizable logic operations and the technology is still far from being a practically applicable technology that can compete with conventional binary logic systems. In this vein, accomplishing some tasks is critical to guide collective research in areas associated with this technology after precise diagnosis of the status of each such research area.

##### Enhancement of the Integration Compatibility

MVL gates apparently have mathematical advantages of increasing the integration level by the higher radix operations. However, the circuit complexity can also increase due to the increased number of interconnections. In this sense, many of the research is focused on developing monolithic multistate devices which can store information in multilevel states of a single device, which can further demonstrate the VLSI compatibility and thus fully harness the mathematical edge of MVLs. To further exploit the advantage of multistate devices, the vertical integration of the component devices can further be employed. Organic semiconductors heterojunctions and 2D vdW heterostructures can be highly benefitted if the current flow through the device is vertically directed, with which the advantage of the vertical transistor architectures such as short channel length, compatibility to the flexible device architecture and the lateral compactness, can be harnessed. Moreover, the vertical integration can further have an advantage in scalability since many of the currently reported approaches are limited by the geometry of the devices, as demonstrated by the lateral overlapping‐area‐dependent fold‐back structures of NDR behaviors.

##### Materials Developments for NDR and NTC Devices

The emergence of chemically versatile organic semiconductors and various 2D materials, along with acquired knowledge of NDR and NTC behaviors, has aided researchers to demonstrate the high feasibility of achieving ideal operation of MVL gates. The ease of fabrication of these devices by solution processes or chemical vapor deposition processes and their suitability for high‐throughput synthesis are highly beneficial for reducing the production cost. However, the electrical properties of organic materials are presently far inferior to those of their inorganic counterparts and the inherently high disorder in their morphology limits further downscaling of devices fabricated using these organic materials. Promisingly, the nonlinearity of electrical characteristics of organic materials can also be achieved via the incorporation of multidimensional nanostructures of these materials. Their ability to be fine‐tuned on account of their chemical versatility is a distinct advantage over other classes of materials. On the other hand, the most commonly researched approach for 2D materials is the fabrication of tunneling diodes using vdW heterostructures of these materials. Their atomically thin structure is a distinct advantage for the achievement and control of tunneling behaviors, but their dimensionality can be a fundamental disadvantage because of inevitably high valley currents. However, such fundamental limitations can be overcome through both modifications of device architectures and adaptation of typical NDR approaches other than tunneling. In particular, various chemical compositions of TMDC materials and the tunability of their energetic and electrical properties can be explored to establish a new series of materials compatible with various NDR mechanisms such as satellite‐valley tunneling and transit time modulations.

##### Materials Developments for QDGFETs

Because of their efficient charge storage properties, 0D QDs are a promising nanostructured material platform for MVL applications. The quantized density of states in QD structures can enable storage of a limited number of charge carriers, which, in turn, will enable generation of a stable intermediate state during QDGFET operation. Furthermore, alternate stacking of QD‐embedded layers enables realization of a system beyond the ternary level (e.g., a quaternary system with two intermediate states). Although such a QD‐assisted approach can provide stable intermediate states and have relevant MVL applications, precise control of the size and spatial distribution of QDs remains a challenge to be overcome for achieving device reliability on a large scale.

In addition to the NDR and NTC behaviors and QDGFET devices, the quantum‐mechanical BTBT mechanism and trap‐induced multistability of spin states of microcavity polaritons have also been successfully used to realize MVL operations. In particular, the BTBT‐based approach has been implemented even on the wafer scale by being applied to the conventional CMOS technology. Although MVL gates have been intensively investigated and developed over the last few decades, several issues in their practical applications remain to be addressed. Therefore, additional research breakthroughs in both theoretical and experimental approaches are required for overcoming the current technological challenges. We believe that after such advances have been made, MVL applications based on emerging materials will usher in the era of hyper Moore's law in the field of electronics.

## Conflict of Interest

The authors declare no conflict of interest.
